# The crosstalk between vascular MSCs and inflammatory mediators determines the pro-calcific remodelling of human atherosclerotic aneurysm

**DOI:** 10.1186/s13287-017-0554-x

**Published:** 2017-04-26

**Authors:** Carmen Ciavarella, Enrico Gallitto, Francesca Ricci, Marina Buzzi, Andrea Stella, Gianandrea Pasquinelli

**Affiliations:** 1 Clinical Pathology-Department of Experimental, Diagnostic and Specialty Medicine (DIMES), S.Orsola-Malpighi Hospital, University of Bologna, Bologna, Italy; 2 Vascular Surgery-Department of Experimental, Diagnostic and Specialty Medicine (DIMES), S.Orsola-Malpighi Hospital, University of Bologna, Bologna, Italy; 3 Immunohematology and Transfusion Service, S.Orsola-Malpighi Hospital, University of Bologna, Bologna, Italy

**Keywords:** Human aortic mesenchymal stem cells, Inflammation, Calcification, Vascular remodelling, Angiogenesis, Atherosclerotic aneurysm

## Abstract

**Background:**

Human mesenchymal stem cells (MSCs) possess well-known reparative abilities, but any defect of the immunomodulatory activity and/or the differentiation process may determine the development of human diseases, including those affecting the vascular wall. MSCs residing within the human aortic wall represent a potential cell mediator of atherosclerotic aneurysm development.

**Methods:**

MSCs isolated from healthy and aneurysm aortas were characterized by flow cytometer and tested for differentiation properties. Healthy aorta (ha)-MSCs were then subjected to inflammatory stimuli to evaluate the microenvironmental impact on their function and involvement in vascular remodelling.

**Results:**

Abdominal aortic aneurysm (AAA)-MSCs were isolated from calcified and inflamed aortas of 12 patients with high serum levels of MMP-9 protein. AAA-MSCs expressed typical mesenchymal markers and, in line with the histological analysis, elevated levels of OPN, an osteogenic marker also involved in vascular remodelling. AAA-MSCs were highly osteogenic and underwent intense calcium deposition under proper stimulation; moreover, AAA-MSCs were able to differentiate into tubule-like structures in Matrigel, even if the lack of CD146 and the reduced structural stability suggested an inefficient maturation process. We further demonstrated an association between osteogenesis and inflammation; indeed, ha-MSCs cultured with either cytokines (TNF-α, IL-1β) or AAA-PBMCs showed increased expression of MMP-9 and osteogenic markers, to the detriment of the adipogenic regulator PPAR-γ. Interestingly, the culture with inflammatory cells highly stimulated ha-MSCs towards the osteogenic commitment.

**Conclusions:**

AAA-MSCs displayed high osteogenic potential and pathological angiogenesis that represent crucial steps for AAA progression; we showed that the inflammatory process critically addresses human vascular MSCs towards a pathological behaviour, inducing vascular bone matrix deposition and remodelling. Inhibition of this pathway may represent a pharmacological approach against arterial calcification.

**Electronic supplementary material:**

The online version of this article (doi:10.1186/s13287-017-0554-x) contains supplementary material, which is available to authorized users.

## Background

The mesenchymal stem cell (MSC) niche has been identified and characterized in several human adult tissues other than bone marrow [1–3]; as many researchers have reported, MSCs are endowed with self-renewal and, under the appropriate stimulation, are able to differentiate into multiple cell types along the mesodermal lineage (adipocytes, chondrocytes, osteocytes) [4]. Thanks to these properties, MSCs regulate many reparative processes in the presence of tissue damage as well as keep the tissue homeostasis under physiologic conditions. Moreover, the low immunogenic profile and the secretion of immunoregulatory molecules (i.e. indoleamine 2,3-dioxygenase (IDO), inteurleukin-10 (IL-10), transforming growth factor beta (TGF-β), human leukocyte antigen-G (HLA-G)) allow MSCs to escape the immune system and suppress the inflammatory process [5, 6]. For these reasons MSCs have been extensively investigated as potential therapeutic strategies [7], although recent findings have introduced novel concepts about MSC behaviour in a pathological context. In particular, MSCs isolated from diseased sources display abnormal characteristics in terms of differentiation and/or immunomodulatory properties, but the mechanisms responsible for these functional alterations are not fully understood [8]. MSCs may be affected by external factors that give rise to a heterogeneous cell population consisting of several cell subsets at distinct stages of proliferation and differentiation [9]. The tissue of origin, the impact of aging and/or inflammation may select MSCs addressed towards a specific lineage commitment, thus contributing to the onset of pathological conditions. Some bone disorders have been associated with defects of the MSC differentiation process; among these, patients affected by osteoporosis showed circulating MSCs with an abnormal osteogenesis [10] and an impaired osteoblastogenesis was also demonstrated in a murine model of osteogenesis imperfecta [11]. Likewise, an uncontrolled osteogenic process may represent a primary mechanism of vascular calcification and increasing evidence supports the direct involvement of vascular cell populations. Indeed, calcium deposition affecting the artery wall is no longer considered a passive and ageing-related event, but rather an active and regulated process involving different cell players [12]; in this regard, the vasculogenic niche of MSCs exerts a critical role. While atherosclerotic calcification has been explored widely, less is known about the presence of calcification in abdominal aortic aneurysms (AAAs), where it seems to be associated with an increased risk of rupture [13]. A patient-specific study revealed that calcification at the adventitia level increases the wall stress, consequently affecting the stability of AAAs [14]. A work by our team described functional abnormalities on a human model of MSCs derived from patients affected by AAA (AAA-MSCs). Consistent with the AAA pathogenesis, AAA-MSCs displayed significant hyper-expression of matrix metalloproteinases (MMPs), especially MMP-9, whereas the reduced release of anti-inflammatory mediators resulted in a weak immunosuppressive effect of AAA-MSCs on activated peripheral blood mononuclear cells (PBMCs) [15]. Vascular remodelling and inflammatory process are tightly connected, because inflammatory mediators stimulate the MMP expression in different biological contexts [16–18]; moreover, a relation has been demonstrated between MMP-mediated elastin degradation and occurrence of calcification in a rat model of AAA [19]. Inflammation may also correlate with vascular calcification and their relationship influences the severity of atherosclerotic disease. Inflammation causes a reduced secretion of osteogenesis inhibitors and induces vascular smooth muscle cells (VSMCs) to secrete matrix vesicles where calcium precipitates, giving rise to calcification nodules [20]. Based on these premises, we hypothesize that MSCs of vascular origin under inflammatory conditions may be addressed towards an aberrant pattern of differentiation and give rise to vascular lesions occurring during AAA development, like calcium deposition, increased neo-vascularization and worsening of the inflammatory process. We analysed the differentiation profile of AAA-MSCs; in order to explore the influence of inflammation on vascular MSCs, we then exposed healthy aortic cells to inflammatory conditions and evaluated their differentiation tendencies.

## Methods

### Study design and patients

The Ethics Committee of University Hospital S. Orsola-Malpighi (Bologna, Italy) approved all procedures included in this study.

Patients candidate for open surgical repair for AAA (12 individuals) were enrolled after written consent; aortic tissues and peripheral blood samples were collected in collaboration with the Unit of Vascular Surgery (S.Orsola-Malpighi University Hospital﻿, Bologna). The calcification extent was measured through non-contrast computed tomography (CT) scored (0–3) according to the extension within the wall.

Healthy tissues belonging to five healthy dead donors and buffy coats obtained from five healthy volunteers were provided by the Cardiovascular Tissue and Cord Blood Bank (S. Orsola-Malpighi University Hospital, Bologna).

### ELISA assay for MMP-9 detection

The serum levels of MMP-9 were determined by enzyme-linked immunosorbent assay (ELISA). Blood samples were collected in a clot activator tube (Vacuette, z-serum beads clot activator) and, after clotting, centrifuged at 2000 × *g* for 10 min. The serum was then aliquoted and stored at −80 °C until analysis. The analysis was performed using the Human MMP-9 Quantikine ELISA kit (DMP900; R&D Systems) according to the manufacturer’s instructions. Serum samples were diluted 100-fold in the calibrator diluent. The optical density of each well was measured by a spectrophotometer at 450 nm within 30 min.

### Immunohistochemistry and Alizarin Red staining

Immunohistochemistry (IHC) was performed on healthy and AAA specimens, using a non-biotin amplified method (Novolink). Briefly, sections (4 μm thick) of formalin-fixed and paraffin-embedded tissues were deparaffinized and rehydrated through a series of graded ethanol and rinsed in distilled water. Endogenous peroxidase activity was blocked in 0.3% H_2_O_2_ in absolute methanol for 10 min at room temperature; antigen retrieval was performed using citrate buffer (pH 6) in a microwave (750 W) for 20 min and, after cooling, slides were washed with Tris-buffered saline (TBS). Aortic sections were subsequently incubated with osteopontin (OPN) primary antibody (1:750; Millipore) in a moist chamber at 4 °C o/n, and then incubated with NovoLink Polymer for 30 min at room temperature and exposed to the substrate/chromogen 3,3′-diaminobenzidine (DAB) prepared from Novocastra DAB Chromogen and NovoLink DAB buffer. Nuclei were counterstained with Mayer’s haematoxylin. Samples were dehydrated, coverslipped and observed under a light microscope (LM) using the Image-Pro Plus program.

Sections (4 μm thick) of healthy and AAA tissues were stained with Alizarin Red to detect calcium deposition. After xylene and alcohol passages, sections were incubated with Alizarin Red solution (pH 4.1–4.3) for 2 min in a moist chamber, dehydrated with acetone 100% and then with 1:1 acetone:xylene, mounted and observed as already described.

### MSC isolation from human healthy aortic and AAA wall

MSCs were isolated from healthy and diseased aortic tissues according to an established enzymatic method [15, 21]. Before digestion, the thrombus and the peri-adventitial adipose tissue were removed from the aneurysm sac. The tissues were then cut into 2-cm^2^ sections and incubated with 0.3 mg/ml Liberase type II (Liberase™ Research Grade; Roche) in serum-free Dulbecco’s modified Eagle’s medium (DMEM; Sigma Aldrich) at 37 °C o/n in a rotor apparatus. The digested tissue was filtered through decreasing diameter cell strainers and centrifuged at 400 x g. After cell viability testing, MSCs isolated from aneurysm (AAA-MSCs) and those obtained from healthy aorta (ha-MSCs) were cultured (37 °C incubator, 5% CO_2_) in DMEM enriched with 20% fetal bovine serum (FBS; Sigma Aldrich) and expanded in vitro. MSCs at passage 3 were analysed by flow cytometer to detect the expression of mesenchymal markers CD44, CD90, CD73 and CD105, as described previously [15].

### Multilineage differentiation assays

In order to test the multilineage differentiation capacities, MSCs at passage 3 were seeded at a density of 20 × 10^4^ cells/well and 10 × 10^4^ cells/well on 12-well plates for adipogenic and osteogenic assay, respectively. After 2 days from seeding, MSCs were exposed to specific induction media according to the manufacturer’s instructions (StemPro Adipogenesis and Osteogenic Differentiation Kit; Life Technologies); untreated MSCs cultured in traditional DMEM with 10% FBS were used as controls. After 14 days, adipogenic-induced cells were in part formalin-fixed and stained with Oil Red O for cytoplasmic lipidic droplet detection, and in part processed for RNA extraction to analyse the adipogenic transcription factor peroxisome proliferator-activated receptor gamma (PPAR-γ).

After 21 days, osteogenic-induced cells were fixed for Alizarin Red staining to detect the mineralization process and processed for RNA extraction to investigate osteogenic marker expression (bone morphogenetic protein-2 (BMP-2), OPN, osteocalcin (OCN)).

### Endothelial differentiation assay

ha-MSCs and AAA-MSCs were exposed to vascular endothelial growth factor (VEGF) at 50 ng/ml for 7 days in DMEM with 2% FBS. Cells grown in DMEM with 2% FBS without VEGF were used as controls. After induction, 15 × 10^3^ cells were seeded onto a semi-solid matrix (Matrigel; BD Biosciences) previously polymerized at 37 °C, 5% CO_2_. MSC morphological changes until the formation of capillary networks were regularly observed starting at 2 hours from seeding and were photographed under light microscopy. The total tubule length, the number of branching points and the number of total tubes in a 10× magnification field were quantified using Wimasis Image Analysis Software (Wimasis GmbH, Munich, Germany). At the end of the sprouting process, neo-vessels were digested with Dispase (1 mg/ml; Sigma Aldrich) for 2 hours at 37 °C and then processed for molecular analysis of CD31, α-smooth muscle actin (α-SMA) and CD146. MSCs were also fixed for flow cytometry analysis before and after Matrigel seeding to analyse CD31 and CD146 proteins.

### MSC exposure to inflammatory conditions

ha-MSCs were subjected to inflammatory stimulation through two different approaches. The first one consisted of co-culture with activated AAA peripheral blood mononuclear cells (AAA-PBMCs). First of all, PBMCs were isolated by density gradient centrifugation from healthy volunteers and from enrolled AAA patients. Healthy (hPBMCs) and AAA-PBMCs were then processed for RNA extraction and compared for cytokine expression. After monocyte adhesion to plastic flask (4 hours), AAA-PBMCs were counted and plated on an MSC feeder layer at a density ratio of 1:2 MSCs:PBMCs in RPMI 1640 (Lonza); 5 μg/ml phytohaemagglutin (PHA; Sigma Aldrich) was added to activate AAA-PBMCs. Co-culture was performed in a transwell culture system, using a 0.4-μm membrane pore size (Corning).

In the second condition, ha-MSCs were exposed to the human recombinant cytokine combination TNF-α and IL-1β (Sigma Aldrich), at a concentration of 25 ng/ml each.

After 24 hours, ha-MSCs primed with inflammatory signals were both processed for RNA extraction and exposed to adipogenic and osteogenic media, as already described.

### Quantitative reverse transcriptase polymerase chain reaction

Total RNA extraction was performed using TRIreagent (TRIzol reagent; Life Technologies) according to the manufacturer’s instructions. One microgram of total RNA was reverse transcribed in a 20-μl reaction volume using a High Capacity Reverse Transcription Kit (Life Technologies). Real-time PCR was carried out in a Gene Amp 7000 Sequence Detection System (Applied Biosystems) using the SYBR green mix (Life Technologies) and specific couples of primers were designed using the NCBI BLAST tool (purchased from Sigma Aldrich; Table 1). Each assay was executed in triplicate and target gene expression was normalized to glyceraldehyde 3-phosphate dehydrogenase (GAPDH). The final results were determined by the comparative 2^–ΔΔCt^ method and expressed as fold changes relative to control.

**Table 1:** Primer sequences

Gene	Primer sequence
α-SMA	FWD: ACCTTTGGCTTGGCTTGTCA REV: GGAAGCTTTAGGGTCGCTGG
BMP-2	FWD: TGTCTTCTAGCGTTGCTGCT REV: CAACTCGAACTCGCTCAGGA
CD31	FWD: CACAGATGAGAACCACGCCT REV: GGCCCCTCAGAAGACAACAT
CD146	FWD: CTCACTCGGACGTCAGACAC REV: AACACAGTGGGCGCTATGAA
GAPDH	FWD: AATGGGCAGCCGTTAGGAAA REV: AGGAGAAATCGGGCCAGCTA
IL-1β	FWD: TGAGCTCGCCAGTGAAATGA REV: AGATTCGTAGCTGGATGCCG
IL-10	FWD: GGGGCTTCCTAACTGCTACA REV: TAGGGGAATCCCTCCGAGAC
MMP-9	FWD: GAACCAATCTCACCGACAG REV: GCCACCCGAGTGTAACCAT
PPAR-γ	FWD: GTGGTAGGTAAGGAAGGGGC REV: GGCTGACTCTCGTTTGAGAA
OPN	FWD: AGGCATCACCTGTGCCATAC REV: GTCCAAGCTTCTGGGGACAA
OCN	FWD: CACCGAGACACCATGAGAGC REV: CTGCTTGGACACAAAGGCTGC
TNF-α	FWD: CAGGGACCTCTCTCTAATCA REV: TTGAGGGTTTGCTACAACAT

*α*-*SMA* alpha-smooth muscle actin, *BMP*-*2* bone morphogenetic protein-2, *CD* cluster of differentiation, *GAPDH* glyceraldehyde 3-phosphate dehydrogenase, *IL* interleukin, *MMP*-*9* matrix metalloproteinase-9, *PPAR*-γ peroxisome proliferation activated gamma, *OPN* osteopontin, *OCN* osteocalcin, *TNF*-α tumour necrosis factor alpha

### Western blot

Total cellular proteins were extracted by MSCs using lysis buffer (0.1 M KH_2_PO_4_, pH 7.5, 1% NP-40, 0.1 mM β-glycerolphosphate, supplemented with protease inhibitor cocktail; Sigma-Aldrich) and quantified spectrophometrically by the Bio-Rad Protein Assay (Bio-Rad Laboratories). Thirty micrograms of proteins were subjected to 8–12% SDS-PAGE and transferred to nitrocellulose membrane (GE Healthcare Life Sciences) at 30 mA for 2 hours and 30 min. Membranes were blocked with 5% non-fat dry milk in TBS-Tween for 1 hour at room temperature and incubated with primary antibodies against OPN (1:500; Millipore), BMP-2 (1:1000; Santa Cruz), PPAR-γ (1:200; Santa Cruz) and β-actin (clone AC-74; Sigma-Aldrich) at 4 °C o/n. Incubation with secondary antibody human anti-rabbit/mouse horseradish peroxidase-conjugated (GE Healthcare) was performed for 1 hour at room temperature. The protein signal was detected using Westar ηC chemiluminescent substrate (Cyanagen) and band intensities were quantified by densitometry analysis by ImageJ software (NIH, USA). Similarly, equal volumes (18 μl) of MSC supernatants, previously cultured in serum-free DMEM for 24 hours and stored at −80 °C, were loaded into 8% SDS-PAGE and transferred to nitrocellulose membrane as already described, using primary MMP-9 antibody (1:1000; Cell Signaling).

### Immunofluorescence

Immunofluorescence was performed on ha-MSCs after osteogenic differentiation to detect MMP-9 protein. Briefly, 3 × 10^4^ cells were seeded on collagen-biocoated slide chambers (BD Bioscence); after 24 hours, cells were washed gently with PBS and fixed with cold absolute methanol, for 10 min at room temperature. Fixed cells were then blocked in 1% bovine serum albumin (BSA) in PBS solution and donkey serum, specific for the secondary antibody species, for 30 min at room temperature. After blocking, cells were incubated with primary MMP-9 antibody (1:500; Cell Signaling) for 1 hour at 37 °C. Samples were then washed with PBS, and incubated with Alexa Fluor 546 (1:250; Invitrogen) secondary antibody in 1% BSA/PBS for 1 hour at 37 °C in the dark. Finally, after washing, the samples were mounted and nuclei were counterstained with Pro Long anti-fade reagent with DAPI (Life Technologies). Images were acquired by a Leica DMI4000 B inverted fluorescence microscope (Leica Microsystems, Milan, Italy).

### Statistical analysis

Each experiment was executed at least in triplicate. Results were analysed by GraphPad Prism 6 statistical software (GraphPad Software Inc.) and expressed as mean ± standard deviation. Statistical analysis was performed using the paired *t* test and one-way and two-way ANOVAs followed by Bonferroni and Tukey’s multiple comparisons test. Pearson’s correlation was used for correlation study. Results were considered statistically significant at the 95% confidence level (*p* < 0.05 was considered significant).

## Results

### AAA calcification burden and MMP-9 levels

**Fig. 1:**
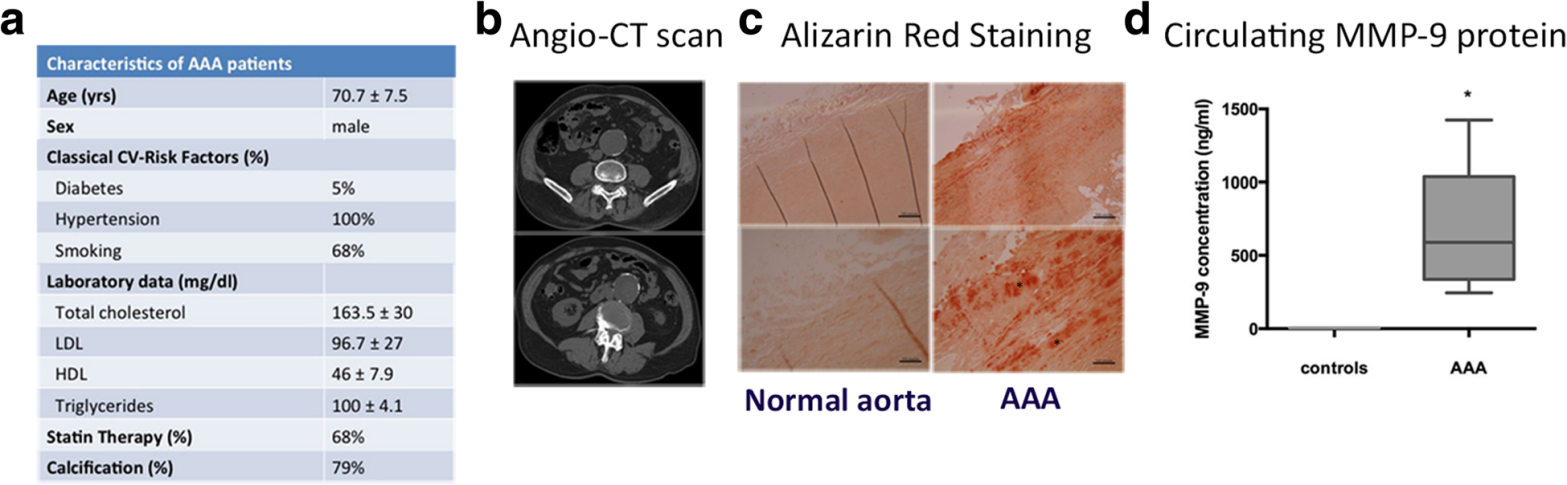
Clinical background of study patients. **a** Clinical data of AAA patients; data are reported as mean ± standard deviation (*n* = 12). **b** Evaluation of calcium content within the aortic wall: representative non-contrast angio-CT acquisition of AAA wall corresponding to scores 1 (*upper panel*) and 3 (*lower panel*). **c** Alizarin Red staining of healthy vs AAA tissue sections showed  the presence of calcium depositions only in the affected aorta (*black stars*), reflecting a diffuse localization within the whole arterial wall with more intensive staining at the media level. **d** Serum levels of MMP-9 protein in AAA patients compared with pooled sera from healthy donors. The assay was executed in duplicate and data are reported as mean ± standard deviation of two independent assays. *p < 0.05. *AAA* abdominal aortic aneurysm, *CV* cardiovascular, *CT* computed tomography, *LDL* low density lipoprotein, *HDL* high density lipoprotein, *MMP-9* matrix metalloproteinase-9

The present study was based on MSCs of vascular origin; in order to contextualize the cellular findings in the clinical background of AAA disease, we summarized the main data referred to the AAA population in Fig. 1a. The evaluation of calcium amount, as assessed by angio-CT scan, reflected the presence of calcification in 79% of the patients (Fig. 1a, b). The presence of calcium deposits was also demonstrated through Alizarin Red staining performed on AAA tissue: as shown in Fig. 1c, an intense positivity to the dye was detected only on pathological samples, at the media level.

To further characterize the patient population, we evaluated the circulating levels of MMP-9 protein, associated with vascular remodelling and inflammation. The serum levels of MMP-9 in AAA patients were significantly higher than in healthy controls (684.7 ± 84 vs 5.5 ± 0.3 ng/ml, *p* < 0.05) as measured by ELISA (Fig. 1d). Interestingly, a significant correlation was found between circulating levels of MMP-9 and the aortic calcification score (*r* ^2^ = 0.38, *p* < 0.05, Pearson’s correlation), suggesting a possible association between the MMP-mediated vascular remodelling and calcification occurrence.

### Vascular MSCs isolated from aneurysm display high multilineage differentiation potential

**Fig. 2:**
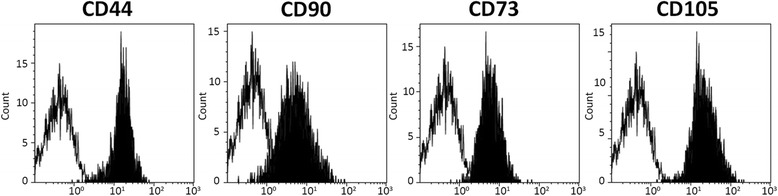
Mesenchymal immunophenotype of AAA-MSCs. Representative images showing flow cytometry for CD44, CD90, CD73 and CD105 confirmed the mesenchymal phenotype of MSCs isolated from the AAA wall (*black histograms*)

**Fig. 3:**
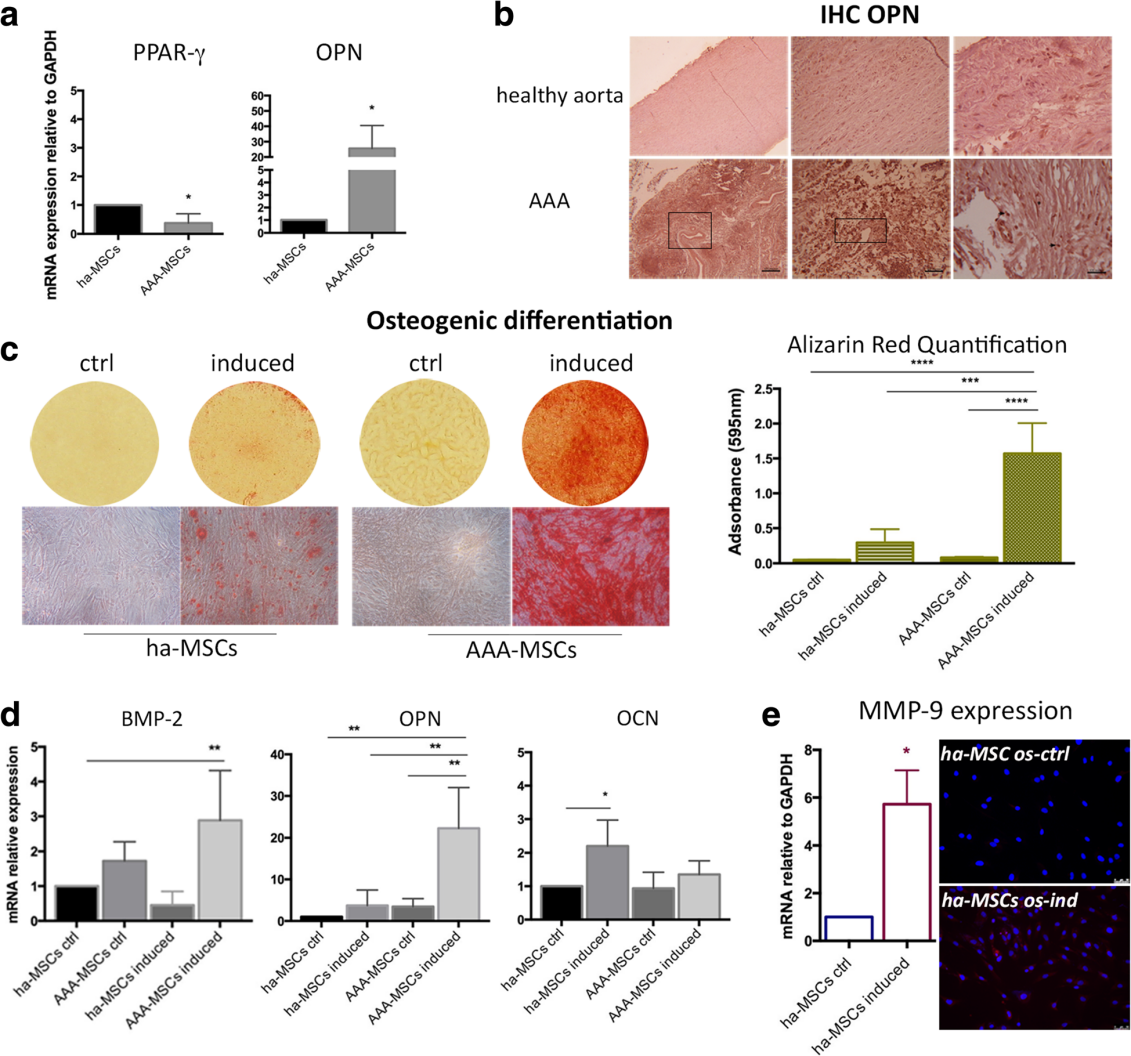
AAA-MSCs possess elevated osteogenic potential. AAA-MSCs were compared with ha-MSCs for expression of lineage-specific markers at time 0 and after stimulation with osteogenic medium. **a** Significant down-regulation of PPAR-γ and pronounced expression of OPN in AAA-MSCs. Results are expressed as fold changes relative to ha-MSCs. **b** In-situ detection of OPN protein was carried out on healthy and aneurysm aortic tissues. Diffuse positivity to OPN was detected on the whole AAA tissue (*boxes*), and predominantly on SMCs and inflammatory cells (*arrows* and *stars*). *Scale bar*: 100 μm. **c** Representative Alizarin Red staining of the mineralization process on ha-MSCs and AAA-MSCs, after exposure to osteogenic induction medium for 21 days. Quantitative data are expressed as mean ± standard deviation and compared with undifferentiated ha-MSCs. **d** Real-time PCR analysis of osteogenic markers in ha-MSCs and AAA-MSCs after osteogenic stimulation. Results are expressed as fold changes relative to undifferentiated ha-MSCs. **e** MMP-9 mRNA and protein detection on ha-MSCs after osteogenic differentiation; increased MMP-9 transcription (6-fold) and staining were observed on differentiated vs undifferentiated ha-MSCs. *Scale bar* 50 μm. *p < 0.05, **p < 0.01, ***p < 0.001, ****p < 0.0001. *BMP-2* bone morphogenetic protein-2, *IHC* immunohistochemistry, *OPN* osteopontin, *OCN* osteocalcin, *PPAR-γ* peroxisome proliferation activated receptor gamma, *ha-MSC* healthy aortic MSC, *AAA-MSC* abdominal aortic aneurysm MSC, *MSC* mesenchymal stem cell, *os-ind* osteogenic-induced, *MMP-9* matrix metalloproteinase-9, *ctrl* control

ha-MSCs and AAA-MSCs at passage 3 were characterized as described previously [15, 22]. Representative images of flow cytometer analysis performed on AAA-MSCs are shown in Fig. 2 and demonstrate a typical mesenchymal immunophenotype (CD44^+^, CD90^+^, CD73^+^, CD105^+^). After characterization, the differentiation potential was evaluated in ha-MSCs and AAA-MSCs at early passages (passages 3–7). At first, a significant increase of OPN gene was reported in AAA-MSCs, whereas the adipogenic PPAR-γ was down-regulated in comparison with ha-MSCs (Fig. 3a). Increased, but not statistically significant, levels of the osteogenic BMP-2 and OCN were also recorded in AAA-MSCs (data not shown). Elevated expression of OPN protein was in situ detected on AAA tissues, especially on smooth muscle cells surrounding the lumen of neo-vessels and chronic inflammatory cells (Fig. 3b).

Cells were cultured in osteogenic medium for 21 days and then stained with Alizarin Red to quantify the intensity of the mineralization process. An enhanced calcium deposition was observed in AAA-MSCs (1.33 ± 0.02 absorbance in induced AAA-MSCs vs 0.16 ± 0.8 in induced ha-MSCs) (Fig. 3c). In parallel, a significant increase of BMP-2 and OPN was observed on differentiated AAA-MSCs in comparison with healthy and AAA controls (Fig. 3d). A remarkable variation  of OCN was observed only in the differentiated ha-MSCs (Fig. 3d). Moreover, significant up-regulation of transcription and enhanced protein expression of MMP-9 were observed in differentiated ha-MSCs (Fig. 3e), suggesting the possible involvement of MMP-9 in the calcification process.

**Fig. 4:**
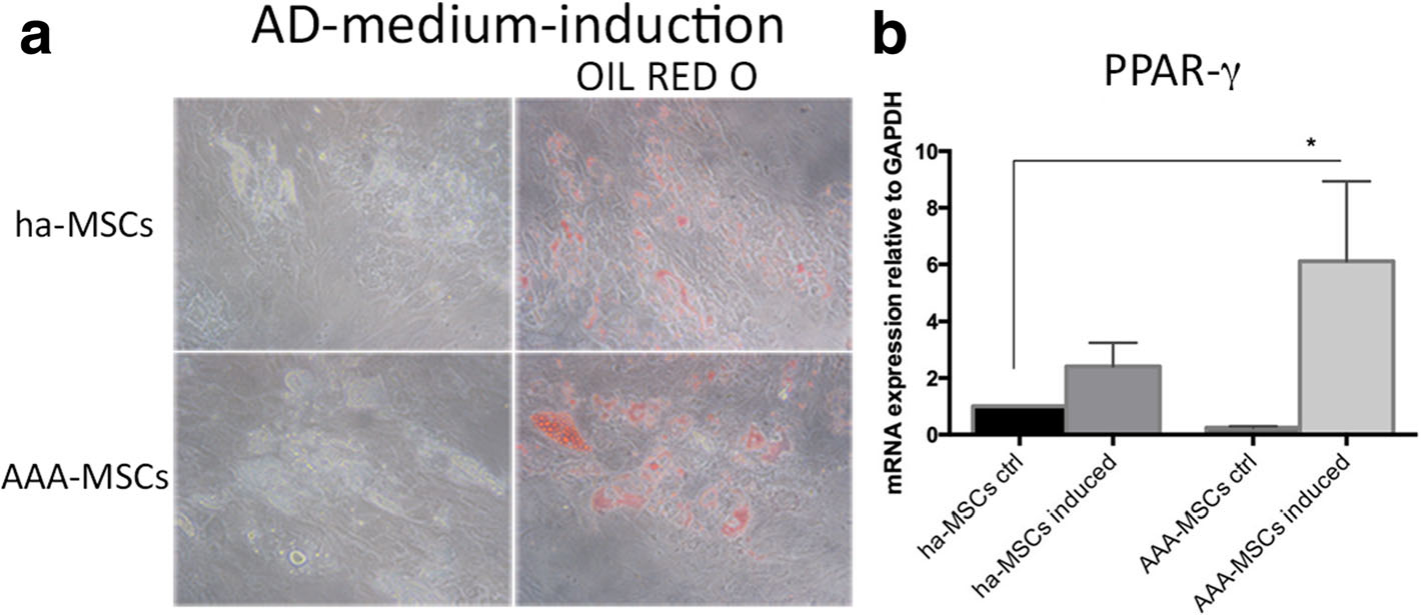
Adipogenic differentiation of AAA-MSCs. **a** Representative Oil Red O staining of ha-MSCs and AAA-MSCs showed the formation of lipid droplets after induction with adipogenic medium for 14 days. **b** Real-time PCR performed on differentiated MSCs revealed significant up-regulation of PPAR-γ in AAA-MSCs following induction. Results are expressed as fold changes relative to undifferentiated ha-MSCs and are representative of at least three independent experiments. *p < 0.05. *AD* adipogenic, *PPAR-γ* peroxisome proliferation activated receptor gamma, *ha-MSC* healthy aortic MSC, *AAA-MSC* abdominal aortic aneurysm MSC, *MSC* mesenchymal stem cell, *ctrl* control

Regarding the adipogenic differentiation, Oil Red O-positive droplets were detected both on healthy and pathological MSCs (Fig. 4a). The higher size of droplets and the enhanced PPAR-γ transcription observed in differentiated AAA-MSCs (6-fold higher compared with ha-MSC control) may suggest an advanced level of differentiation (Fig. 4b).

### AAA-MSCs possess impaired angiogenic potential

**Fig. 5:**
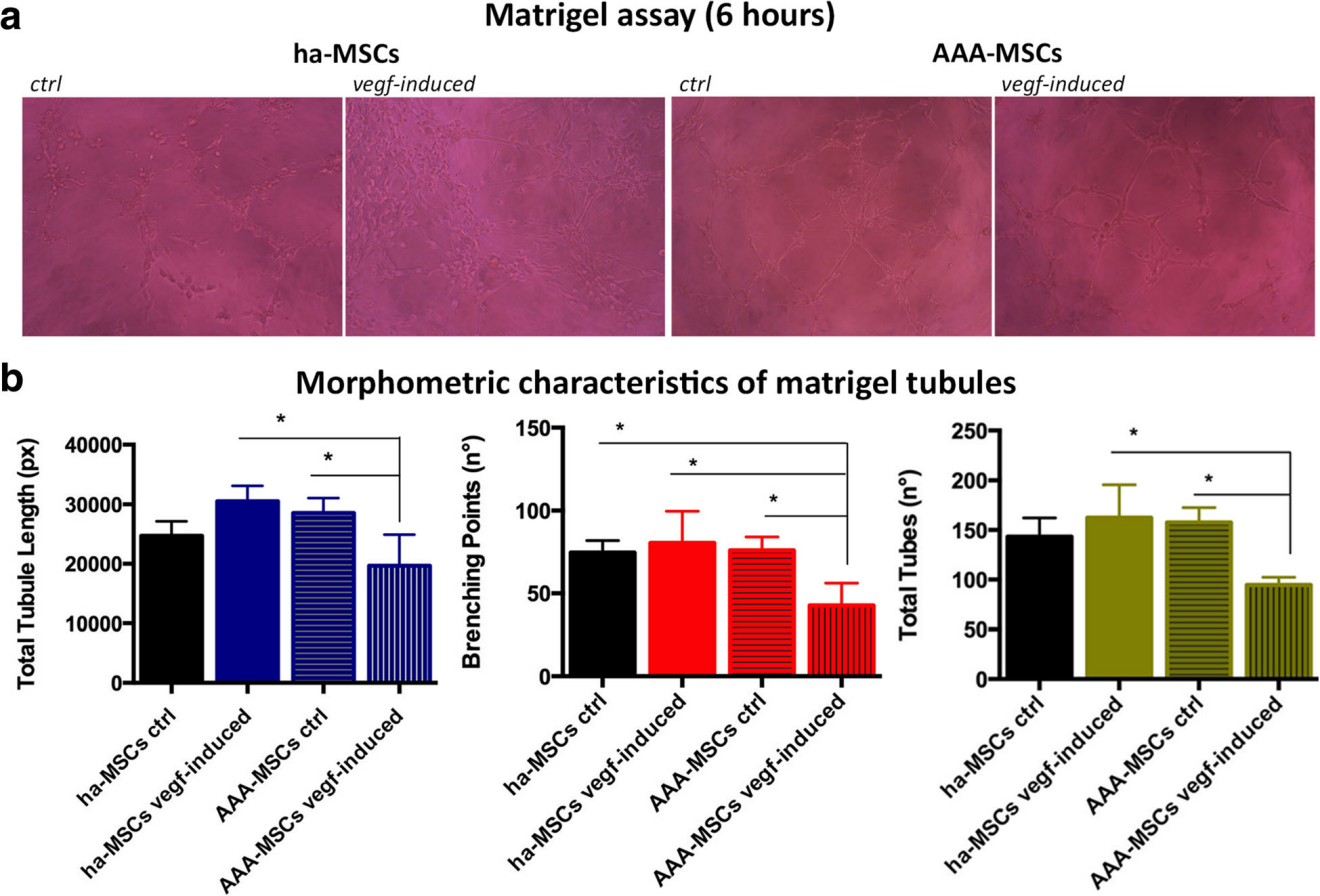
Impaired endothelial differentiation in AAA-MSCs. **a** Representative pictures of ha-MSCs and AAA-MSCs after culture in Matrigel for 6 hours. *Scale bar*: 50 μm. **b** Morphometric analysis of neo-vessels performed on Matrigel-differentiated MSCs after 6 hours, according to total tubule length and number of branching points and total tubes (Wimasis Image Analysis software). Results are expressed as fold changes relative to ha-MSC control; statistical analysis was performed by two-way ANOVA with multiple comparisons among all experimental conditions. *p < 0.05. *ha-MSC* healthy aortic MSC, *AAA-MSC* abdominal aortic aneurysm MSC, *MSC* mesenchymal stem cell, *ctrl* control, *VEGF* vascular endothelial growth factor

**Fig. 6:**
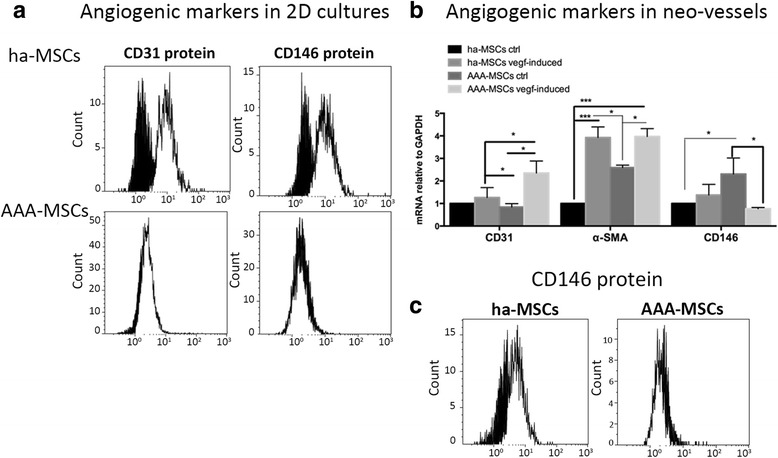
Low expression of CD146 in AAA-MSC newly formed vessels. **a** Flow cytometry analysis of CD31 and CD146 proteins in ha-MSC and AAA-MSC 2D culture before seeding in Matrigel. **b** Gene expression profiles of angiogenic markers CD31, α-SMA and CD146 in healthy and AAA-MSCs after Matrigel culture for 6 hours. Results are expressed as fold changes relative to ha-MSC control; statistical analysis performed by two-way ANOVA with multiple comparisons among all experimental conditions. *p < 0.05, ***p < 0.001. **c** CD146 protein expression in ha-MSC and AAA-MSC newly formed vessels, as detected by flow cytometry after Matrigel dissociation. **a**, **c** *Black histograms*, untreated ctrl; *white histograms*, VEGF-induced condition. *ha-MSC* healthy aortic MSC, *AAA-MSC* abdominal aortic aneurysm MSC, *MSC* mesenchymal stem cell, *ctrl* control, *VEGF* vascular endothelial growth factor

Pathological angiogenesis, associated with the formation of small and unstable vessels, represents a further critical event associated with the risk of aneurysm rupture. In this study, we analysed the morphometric and molecular characteristics of MSCs after endothelial differentiation. Healthy and AAA-MSCs were exposed to VEGF for 7 days and then seeded in Matrigel; as shown in Fig. 5a, both ha-MSCs and AAA-MSCs were able to differentiate into tubule-like structures after 6 hours of culture in Matrigel. Nevertheless, VEGF-induced AAA-MSCs showed a lower structural stabilization as demonstrated by reduced quantification of the total tubule length and the number of branching points and total tubes (Fig. 5b). Indeed, VEGF did not significantly improve the expression of CD31 and CD146 angiogenic markers in AAA-MSCs (Fig. 6a); moreover, AAA-MSC neo-vessels post Matrigel dissociation showed higher levels of CD31 and α-SMA transcription, whereas CD146 underwent a significant decrease both at the mRNA and protein levels (Fig. 6b, c). These data suggest that MSCs may promote the formation of immature vascular networks under pathological conditions, further contributing to the instability and rupture of aneurysm. Conversely, ha-MSC angiogenesis was improved by VEGF stimulation, as reflected by the angiogenic marker expression (Fig. 6a–c).

AAA-MSCs were further analysed for the angiogenic marker expression before and after the Matrigel culture. CD31 and α-SMA levels were not significantly affected, whereas CD146 mRNA underwent a drastic decrease (2.3 ± 0.7 Matrigel AAA-MSC control and 0.8 ± 0.01 Matrigel AAA-MSC VEGF induced vs 30 ± 3.7 and 32.2 ± 6.3 in AAA-MSC control and AAA-MSC VEGF induced, respectively; *p* < 0.0001, two-way ANOVA with Tukey’s post test) (Additional file 1: Figure S1).

### Inflammatory mediators stimulate ha-MSCs towards a pathological phenotype

**Fig. 7:**
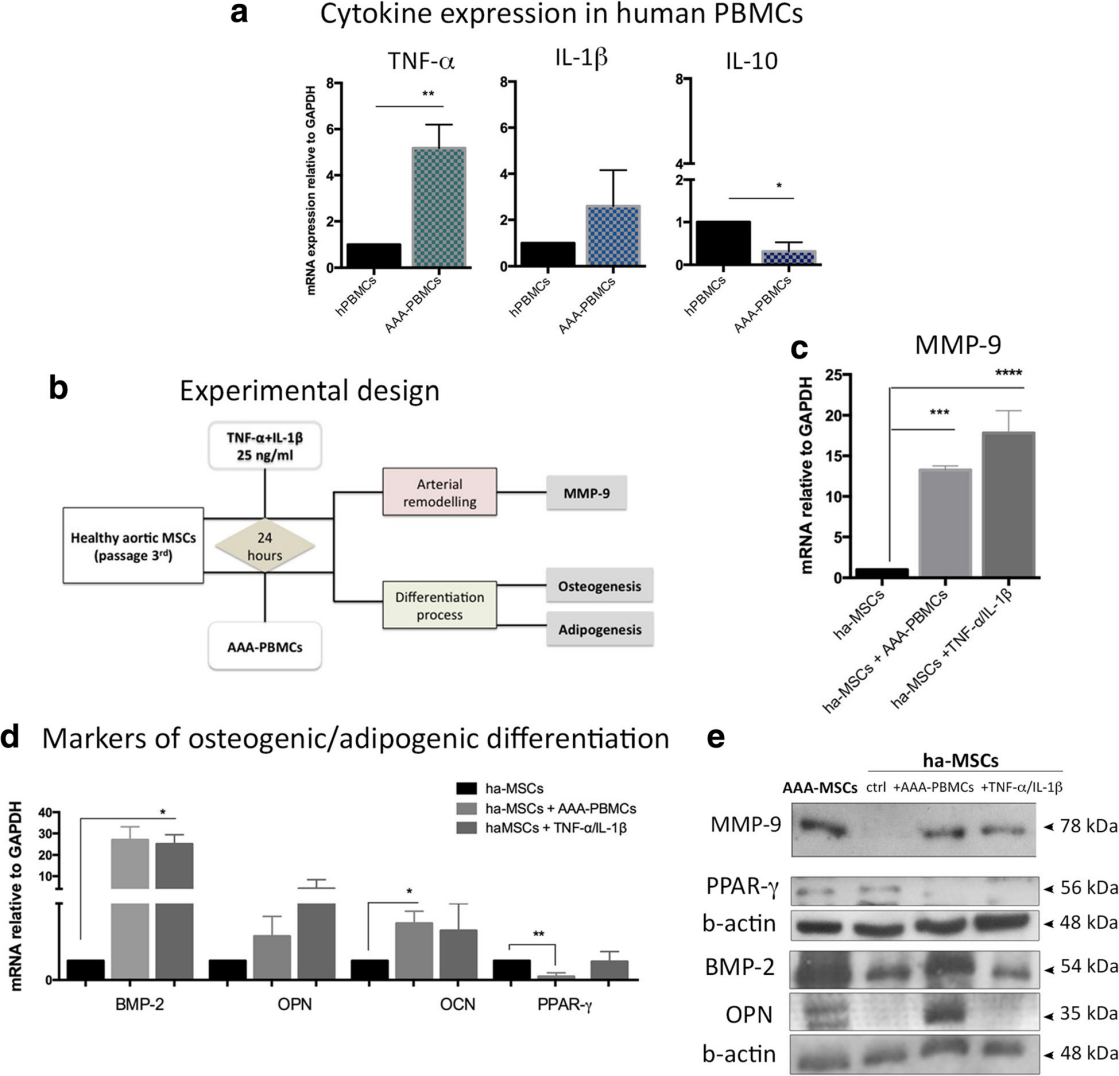
ha-MSCs exposed to inflammatory conditions assume a pathological phenotype. **a** PBMCs isolated from AAA patients showed a molecular signature reporting high levels of inflammatory cytokines (TNF-α and IL-1β) and reduced anti-inflammatory IL-10. Results are expressed as fold changes relative to healthy PBMCs. **b** According to the experimental design, ha-MSCs were exposed to inflammatory mediators (cytokines and PBMCs) for 24 hours, then investigated in terms of vascular remodelling and differentiation properties. ha-MSCs exposed to inflammation underwent increased transcription of (**c**) MMP-9 and (**d**) osteogenic lineage-specific markers (BMP-2, OPN, OCN), to the detriment of the adipogenic transcriptional factor PPAR-γ. Results are expressed as fold changes relative to unexposed ha-MSCs. *p < 0.05, **p < 0.01, ***p < 0.001, ****p < 0.0001. **e** Western blot analysis of MMP-9 was performed on serum-free conditioned media, whereas PPAR-γ, BMP-2 and OPN were detected in cell lysates. *PBMC* peripheral blood mononuclear cell, *TNF-α* tumour necrosis factor alpha, *IL* interleukin, *ha-PMSC* healthy aortic PMSC, *AAA-PMSC* abdominal aortic aneurysm PMSC, *MSC* mesenchymal stem cell, *MMP-9* matrix metalloproteinase-9, *BMP-2* bone morphogenetic protein-2, *OPN* osteopontin, *OCN* osteocalcin, *PPAR-γ* peroxisome proliferation activated receptor gamma, *ctrl* control

In order to investigate the effect of inflammation on vascular MSCs, we tested two different inflammatory conditions. Considering the high amount of inflammatory cells infiltrating the aneurysm wall, the first condition consisted of the interaction with activated immune cells, whereas the second one was based on the exposure to a combination of recombinant inflammatory cytokines TNF-α/IL-1β at 25 ng/ml each. PBMCs were collected from AAA patients (AAA-PBMCs) to better reproduce the in vivo condition. AAA-PBMCs displayed an inflammatory molecular signature (Fig. 7a), with an advanced activation state in comparison with healthy PBMCs (data not shown).

The experimental design is illustrated in Fig. 7b. After exposure to inflammation, ha-MSCs were analysed for MMP-9 and differentiation markers. MMP-9 mRNA increased of 13- and 18- fold in ha-MSCs after exposure to AAA-PBMCs and inflammatory cytokines, respectively (Fig. 7c). Moreover, osteogenic markers were up-regulated in response to inflammation. Conversely, a significant reduction of PPAR-γ transcription was reported after culture with AAA-PBMCs (Fig. 7d), suggesting a molecular switching of ha-MSCs into the osteogenic program despite the adipogenic lineage. Western blot analysis performed on MSC supernatants reported an increased amount of secreted MMP-9 (Fig. 7e) and higher protein levels of osteogenic markers BMP-2 and OPN to the detriment of PPAR-γ in cell lysates following inflammatory stimulation (Fig. 7e).

### Inflammatory priming of ha-MSCs is associated with enhanced osteogenic commitment

**Fig. 8:**
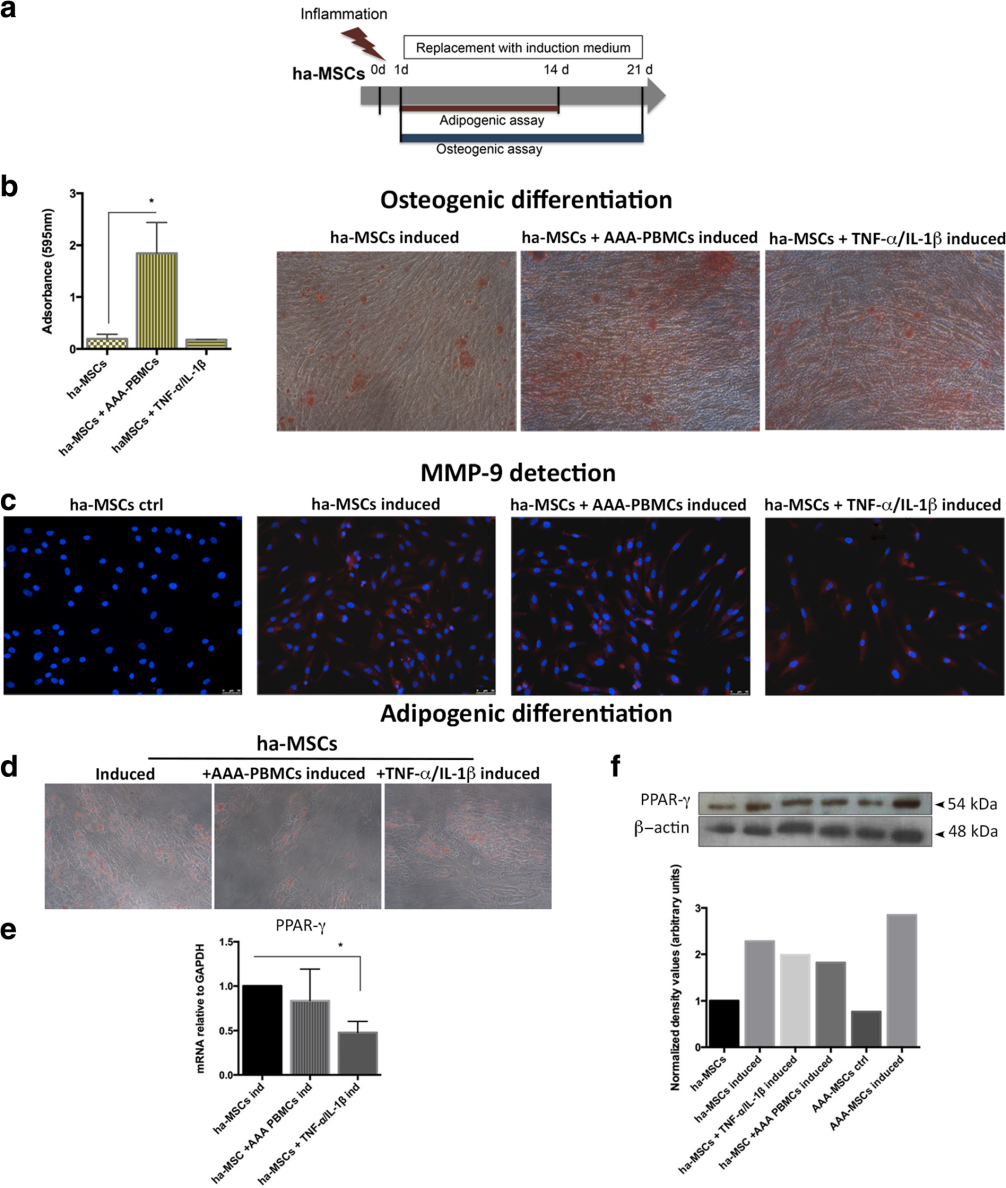
Inflammation enhances the mineralization process in ha-MSCs. **a** After inflammatory stimulation, ha-MSCs were cultured with specific osteogenic and adipogenic induction media for 21 and 14 days, respectively. **b** Calcium mineralization process was significantly marked under inflammatory conditions, mainly after AAA-PBMC influence as showed by Alizarin Red staining. Quantification values are represented as mean ± standard deviation and compared with induced ha-MSCs. **c** MMP-9 detection on osteogenic differentiated ha-MSCs was performed by immunofluorescence, revealing an appreciable staining only after osteogenic induction; 20× magnification. **d** Oil Red O staining of lipid droplets in ha-MSCs was reduced after priming cells with inflammatory cytokines, as shown by **e** PPAR-γ mRNA. Results expressed as fold changes relative to induced ha-MSCs. *p < 0.05. **f** Representative western blot analysis of PPAR-γ protein and relative densitometry after β-actin normalization (ImageJ software). *PBMC* peripheral blood mononuclear cell, *TNF-α* tumour necrosis factor alpha, *IL* interleukin, *ha-MSC* healthy aortic MSC, *AAA-MSC* abdominal aortic aneurysm MSC, *MSC* mesenchymal stem cell, *MMP-9* matrix metalloproteinase-9, *PPAR-γ* peroxisome proliferation activated receptor gamma, *ctrl* control

After stimulation with inflammatory mediators, we analysed the differentiation abilities of ha-MSCs (Fig. 8a). As showed by Alizarin Red staining (Fig. 8b), the mineralization process increased after 24-hour cell priming with inflammatory stimuli; in fact, enhanced calcium amounts were detected on ha-MSCs treated with the inflammatory cytokine combination and in a significant manner after co-culture with AAA-PBMCs. In these conditions, higher expression of MMP-9 protein was observed (Fig. 8c). Adipogenic differentiation was slightly affected, with a lower degree of Oil Red O-positive droplets on ha-MSCs (Fig. 8d) and a decrease of PPAR-γ transcript (Fig. 8e); the protein expression was not drastically influenced (Fig. 8f).

## Discussion

MSCs possess undisguised reparative properties, even if in the presence of functional alterations they may become cell players of disease progression. The fate and behaviour of MSCs are the results of several factors, including the microenvironment, the tissue source and interplay with the surrounding cells. More specifically, MSCs belonging to distinct tissues possess differential properties in terms of proliferation, differentiation abilities and immunomodulation. According to the tissue of origin, MSCs can be more susceptible of alterations or undergo an aberrant differentiation pattern favouring the onset of pathological conditions. This is true for different pathological contexts, especially inflammatory and degenerative affections; the implications of the inflammatory process, which arises in combination with ageing and during several human diseases, on MSC behaviour are reaching growing interest among researchers, but controversial and poor data are currently available. The effect of inflammatory cytokines has been studied on bone marrow and adipose MSCs, demonstrating the maintenance of cell morphology and, in some cases, the improvement of the immunomodulatory efficacy [23, 24]. Meanwhile, studies on aged mice demonstrated that bone marrow MSCs were less effective regarding their activation potential and immunomodulation, following a decreased cytokine expression [25]. The pro-inflammatory cytokine TNF-α improved osteogenic differentiation on pre-differentiated bone marrow MSCs, as demonstrated in a study by Croes et al. [26].

The main goal of our study was to investigate the relationship between vascular MSCs and inflammation, focusing on the differentiation abilities contextual to AAA pathology. AAA represents a common age-related disease, mainly affecting males older than 65 years, whose pathogenesis is extremely complex and not yet associated with a specific aetiological factor [27]. Many studies indicate that the inflammatory process plays a determining role, since the high affluence of inflammatory cells and soluble mediators increase the vessel wall instability, inducing the parietal cell populations to continuously express and release MMPs [28]. In this regard, the vasculogenic niche of MSCs has been demonstrated to be an active part of the AAA pathogenic mechanisms.

The calcification process has been recognized as a degenerative factor common to many inflammatory arterial diseases, but its role in AAA progression is not completely elucidated and few studies have addressed this issue [13]. A Recent work on a murine model of atherosclerosis demonstrates the involvement of vascular progenitor cells in aortic atherosclerosis as well as in ectopic calcification, under inflammatory conditions [29]. Even the genetic ablation of adventitial Gli1^+^MSC-like cells in atherosclerotic mice affected by chronic kidney disease drastically decreases the level of vascular calcification [30]. Indeed the vascular mineralization is complex, based on the active participation of cell components as well as an unbalanced ratio between osteogenic inducers and inhibitors [31]. In the present study, we demonstrate for the first time that MSCs derived from the AAA wall possess a pronounced tendency for osteogenic commitment.

AAA-MSCs exhibited an efficient differentiation degree under the appropriate stimulation; the enhanced osteogenic differentiation represents an intriguing finding in addition to the uncontrolled MMP-mediated matrix degradation and the sharpening of inflammation, not regulated by vascular MSCs. As confirmed by the molecular analysis of the osteogenic markers on terminally differentiated MSCs, BMP-2 and OPN were significantly up-regulated. Therefore, these conditions may favour the occurrence of structural alterations within the aortic wall, undergoing excessive ECM degradation and calcific matrix deposition. Hyper-expression of OPN was also found at the protein level and high amounts of OPN-positive cells were detected on diseased aortic wall, in part on inflammatory cells. Extensive data demonstrate that OPN is not just involved in bone remodelling and vascular calcification, but also regulates vascular proteolysis and correlates with AAA diameter and progression [32]. Moreover, OPN stimulates angiogenesis under VEGF induction [33]; in this view, OPN can be a critical mediator of the complex interplay occurring between inflammatory cells and vascular progenitors.

As documented in literature, the increase of small neo-vessels contributes to AAA progression and represents a risk for rupture [34]. IHC analysis performed on AAA sections demonstrated the expression of OPN on SMCs surrounding the AAA neo-vessels. According to the hypothesis of Collett and Canfield, angiogenesis and vascular calcification correlate [35]: a possible mechanism driving this association derives from the adventitial neo-vessel invasion in the media and intima layers, allowing the passage of vascular progenitors/pericytes that undergo osteoblastogenic differentiation. These conditions are strongly influenced by inflammation. Our study demonstrates that vascular MSCs participate in the aneurysm angiogenesis through the formation of small and immature vessels. In fact AAA-MSCs cultured in Matrigel, the later stage of endothelial differentiation, underwent a drastic decrease of CD146, especially in the presence of VEGF. The morphometric analysis also showed a lower stability of neo-vessels, characterized by reduced branching points, shorter tubule length and fewer total tubes; altered or incomplete neo-vessels can be more prone to rupture and finally contribute to aneurysm instability. Inflammation definitely plays a pivotal role in many pathological processes affecting the aortic wall; in this regard, our data also demonstrate that ha-MSCs in the presence of an inflammatory environment underwent increased expression of MMP-9 and osteogenic mediators. Further confirmation comes from the differentiation assays, showing higher mineralization activity under AAA-PBMC influence. These data support the role of vascular MSCs in the development of arterial calcification, in accordance with the literature on MSCs obtained from animal models [29, 30].

## Conclusions

Despite the low number and the elevated mean age of AAA patients, the present study introduces novel findings, showing first evidence for the crosstalk between human aortic MSCs and inflammatory mediators; moreover, an association can be proposed among inflammation, matrix remodelling and neo-angiogenesis coordinated by MMP-9 and OPN. More interestingly, we found that MMP-9 correlates with calcium scoring in the AAA population; in this regard, future studies will be aimed at increasing the study sample. The involvement of MMP-9 not just in matrix degradation but also in the calcification process can be hypothesized, since we observed increased levels in osteogenic differentiated MSCs. In this view, MMP-9 inhibition could represent a novel approach to reduce ectopic calcification, preventing aneurysm rupture.

## Additional file

Additional file 1:
**Figure S1.** Angiogenic markers in AAA-MSCs before and after culture in matrigel. Significant decrease of CD146 mRNA can be observed after AAA-MSC differentiation in Matrigel; statistical differences were performed by two-way ANOVA with multiple comparisons among all experimental conditions. *****p* < 0.0001. (TIF 1969 kb)

## Abbreviations

AAA-MSC: Abdominal aortic aneurysm mesenchymal stem cell
DMEM: Dulbecco’s modified Eagle’s medium
FBS: Fetal bovine serum
ha-MSC: Healthy aortic mesenchymal stem cell
IL: interleukin
MMP-9: Matrix metalloproteinase-9
OCN: Osteocalcin
OPN: Osteopontin
PBMC: Peripheral blood mononuclear cell
PBS: Phosphate-buffered saline
PHA: Phytohaemagglutin
PPARγ: Peroxisome proliferator-activated receptor gamma
PCR: Polymerase chain reaction
RT: Reverse transcriptase TNF-α, Tumour necrosis factor alpha
VEGF: Vascular endothelial growth factor
