# Supplementary figures and images for: The crosstalk between vascular MSCs and inflammatory mediators determines the pro-calcific remodelling of human atherosclerotic aneurysm
Source: Stem Cell Res Ther. 2017 Apr 26;8:99. doi: 10.1186/s13287-017-0554-x (PMC5406974; doi:10.1186/s13287-017-0554-x)

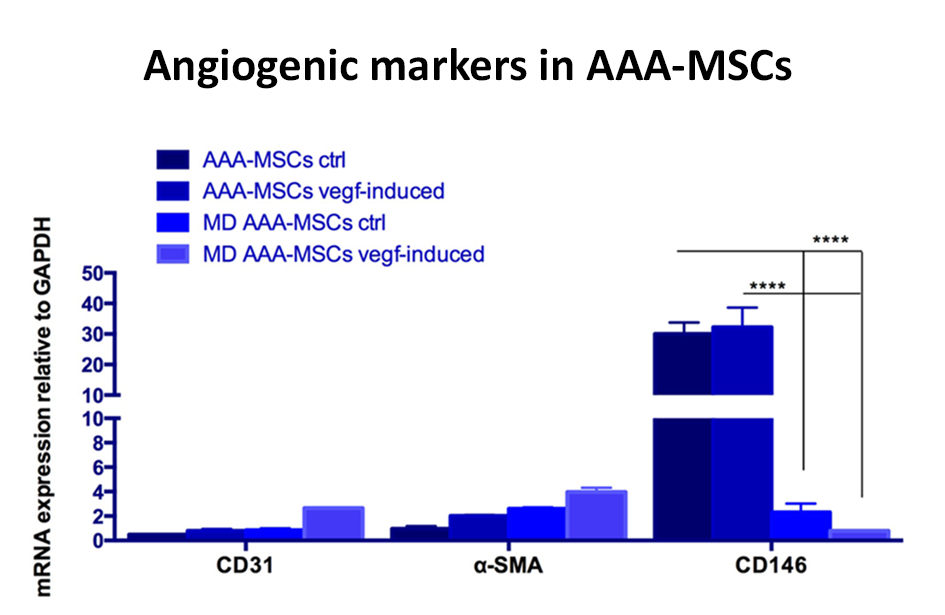

Supplement: Additional file 1: — Figure S1. Angiogenic markers in AAA-MSCs before and after culture in matrigel. Significant decrease of CD146 mRNA can be observed after AAA-MSC differentiation in Matrigel; statistical differences were performed by two-way ANOVA with multiple comparisons among all experimental conditions. ****p < 0.0001. (TIF 1969 kb) [file 13287_2017_554_MOESM1_ESM.tif]
